# Supplementary material for: GeoSES: A socioeconomic index for health and social research in Brazil
Source: PLoS One. 2020 Apr 29;15(4):e0232074. doi: 10.1371/journal.pone.0232074 (PMC7190143; doi:10.1371/journal.pone.0232074)
Supplement: S1 File — (PDF) [file pone.0232074.s001.pdf]

GeoSES: um índice socioeconômico para estudos em saúde no Brasil

Resumo

As condições socioeconômicas do indivíduo são as mais relevantes para prever a qualidade de sua saúde. No entanto, essas informações geralmente não são encontradas nos prontuários médicos, dificultando os estudos na área. Portanto, é comum o uso de índices únicos que caracterizam uma região socioeconômica, como o Índice de Desenvolvimento Humano (IDH). A principal vantagem do IDH é sua compreensão e adoção em escala global. Todavia, sua aplicabilidade é limitada em regiões de menor granularidade (como estados e municípios), pois não contempla as múltiplas dimensões que caracterizam as diferenças socioeconômicas. Aqui apresentamos o GeoSES, um índice composto que resume as principais dimensões do contexto socioeconômico brasileiro para fins de pesquisa. Criamos o índice a partir do Censo Brasileiro de 2010, cuja seleção das variáveis foi orientada por referenciais teóricos para estudos em saúde, incorporando sete dimensões socioeconômicas: educação, mobilidade, pobreza, riqueza, renda, segregação e privação de recursos e serviços. Desenvolvemos o GeoSES usando Análise de componentes principais e avaliamos sua construção, conteúdo e aplicabilidade. O GeoSES é definido por três escalas: nacional (GeoSES-BR), Unidade Federativa (GeoSES-FU) e intra-municipal (GeoSES-IM). As dimensões do GeoSES-BR mostraram boa associação com o IDH-M (acima de 0,85). O modelo com a dimensão pobreza explicou melhor o risco relativo de mortalidade por causas evitáveis no Brasil. Na escala intra-municipal, o modelo com GeoSES-IM foi o que melhor explicou o risco relativo de mortalidade por doenças do aparelho circulatório. Demonstramos que o GeoSES apresenta um potencial explicativo significativo nas

escalas estudadas sendo um complemento convincente para futuras pesquisas em saúde pública.

Palavras-chave

Fatores Socioeconômicos, Indicadores (Estatística), Determinantes Sociais da Saúde, Análise Espacial, Censos
