# Supplementary material for: GeoSES: A socioeconomic index for health and social research in Brazil
Source: PLoS One. 2020 Apr 29;15(4):e0232074. doi: 10.1371/journal.pone.0232074 (PMC7190143; doi:10.1371/journal.pone.0232074)
Supplement: S1 Appendix — (DOCX) [file pone.0232074.s002.docx]

# Appendix 1

Input variables for creating GeoSES

| **VARIABLE** | **MEANING** |
| --- | --- |
| **“Education” Dimension** | |
| **P_GRAD** | Percentage of people for whose kind of the highest completed degree was higher education |
| **P_MEST** | Percentage of people for whose kind of the highest completed degree was master |
| **P_DOUTOR** | Percentage of people whose kind of the highest completed degree was doctorate |
| **P_SEM_INST** | Percentage of people whose level of education is unschooled or incomplete Primary school |
| **P_FUND** | Percentage of people whose level of education is complete primary school and incomplete high schoolo |
| **P_ENSMED** | Percentage of people whose level of education is complete high school and incomplete higher education |
| **P_ENSSUP** | Percentage of people whose level of education is complete higher education |
| **“Mobility” Dimension** | |
| **P_OUTROMUNC** | Percentage of people working in another municipality |
| **P_CASADIA** | Percentage of people returning home from work daily |
| **P_ATE5** | Percentage of people whose usual time spent commuting from home to work is up to 5 minutes |
| **P_6A30** | percentage of people whose usual time spent commuting from home to work is up to 6 to 30 minutes |
| **P_1A2** | percentage of people whose usual time spent commuting from home to work is 1-2 hours |
| **P_MAISDE2** | percentage of people whose usual time spent commuting from home to work is more than 2 hours |
| **“Poverty” Dimension** | |
| **MEDIA_DENSMORA** | resident density per room |
| **P_POBREZA** | % of people in poverty line: whose per capita household income per month is less than or equal to R$ 255.00 or US$144.89 (half minimum wage in 2010) |
| **P_PPI_POBREZA** | % of people in the poverty line and race, black, brown or indigenous |
| **P_BOLSA_FAM** | percentage of people who in July 2010 had a regular monthly income from the Bolsa Familia Social Program or the Child Labor Eradication Program (PETI) |
| **P_OUTROSPROG** | percentage of people who in July 2010 had regular monthly income from other social programs or transfers |
| **“Material deprivation” Dimension** | |
| **P_ALVSREV** | Percentage of homes with uncoated masonry |
| **P_REDE_ESG** | Percentage of households with general sewerage |
| **P_REDE_AGUA** | Percentage of households with general water distribution network |
| **P_LIXO** | Percentage of households with garbage collected directly by cleaning service |
| **P_ENERGIA** | Percentage of households with electricity from electricity distribution company |
| **P_TV** | Percentage of households with TV |
| **P_MAQLAV** | Percentage of households with washing machine |
| **P_GELADEIRA** | Percentage of households with refrigerator |
| **P_MAQTVGEL** | Percentage of households with washing machine, TV and refrigerator |
| **P_CELULAR** | Percentage of households with cell phones |
| **P_COMP_INT** | Percentage of households with computer with internet access |
| **P_CELCOMPINT** | Percentage of households with mobile phone and internet computer |
| **P_MOTO** | Percentage of households with motorcycle for private use |
| **P_CARRO** | Percentage of households with private car |
| **P_ADEQ** | Percentage of households with adequate housing |
| **P_TUDOADEQ** | Percentage of households with access to sewerage, water supply, garbage collection, electricity and adequate housing |
| **P_NEM_MOTO_CARRO** | Percentage of households without motorcycles or cars ownerships for private use |
| **P_SO_MOTO** | Percentage of households with only motorcycles ownership for private use |
| **P_SO_CARRO** | Percentage of households with only private car ownership |
| **“Income” Dimension** | |
| **MED_RENDDOM** | monthly household income in july 2010, in Brazilian Reais |
| **“Wealth” Dimension** | |
| **P_ALUG1000** | percentage of rented households with rental value of R$1,000.00 (US$ 568.20) or more |
| **P_BANH4OUMAIS** | Percentage of households with 4 or more bathrooms |
| **P_IDOSO10SM** | % of people aged 65 years and over with a monthly income equal to or above R$ 5,100.00 (US$ 2,897.72 or 10 Brazilian minimum wages) |
| **“Segregation” Dimension** | |
| **ICE_renda** | (number of people with income above R$ 5,400.00 - number of people with income below R$ 1,000.00) / number of respondents [figures were calculated based on the 20 and 80 percentiles of income V6529 in the PERSON spreadsheet 2010 Census microdata] |
| **ICEedu** | (Number of persons with completed higher education - Number of persons without education and incomplete elementary school)/Total respondents [V6400] |
| **ICE_renda_preto** | (number of whites with income over R$ 5,400.00 - number of blacks with income equal to or less than R$ 1,000.00) / total number of people who answered both questions [V6529 and V0606] |
| **ICE_renda_ppi** | (number of whites with income over R$ 5,400.00 - number of black + brown + indigenous with income equal to or less than R$ 1,000.00) / total number of people who answered both questions [V6529 and V0606] |
| **ICE_branco_renda** | (number of whites with income over R$ 5,400.00 - number of whites with income equal to or less than R$ 1,000.00) / total number of people who answered both questions [V6529 and V0606] |
