# Supplementary material for: GeoSES: A socioeconomic index for health and social research in Brazil
Source: PLoS One. 2020 Apr 29;15(4):e0232074. doi: 10.1371/journal.pone.0232074 (PMC7190143; doi:10.1371/journal.pone.0232074)
Supplement: S1 Table — (DOCX) [file pone.0232074.s005.docx]

**S1 Table.** Geographically Weighted Regression results of models for relative risk of causes of deaths (from 5 to 74 years old) in Brazil due to interventions at the Brazilian Health System in Brazil (2013 to 2017).

| **Model** | **Residual**  **Squares** | **Effective**  **Number** | **Sigma** | **AICc** | **R^2^** | **R^2^**  **Adjusted** |
| --- | --- | --- | --- | --- | --- | --- |
| Model 1  HDI-M | 21.64 | 129.72 | 0.17 | -524.22 | 0.58 | 0.50 |
| Model 2  HDI-M/education | 110.19 | 694.01 | 0.16 | -3,817.45 | 0.49 | 0.41 |
| Model 3  HDI-M/longevity | ~ | ~ | ~ | ~ | ~ | ~ |
| Model 4  HDI-M/income | 30.75 | 195.51 | 0.16 | -1,017.92 | 0.60 | 0.53 |
| Model 5  GeoSES-IM | 114.59 | 746.62 | 0.15 | -4,583.44 | 0.50 | 0.42 |
| Model 6  GeoSES-IM/education | 80.00 | 542.89 | 0.15 | -3,290.28 | 0.51 | 0.43 |
| Model 7  GeoSES-IM/poverty | 111.11 | 737.58 | 0.15 | -4,702.75 | 0.51 | 0.44 |
| Model 8  GeoSES-IM/deprivation | 113.65 | 736.07 | 0.15 | -4,648.99 | 0.50 | 0.43 |
| Model 9  GeoSES-IM/wealth | 118.83 | 766.41 | 0.16 | -4,344.18 | 0.48 | 0.40 |
| Model 10  GeoSES-IM/income | 113.14 | 755.10 | 0.15 | -4,636.58 | 0.51 | 0.43 |
| Model 11  GeoSES-IM/segregatio | 115.72 | 702.04 | 0.16 | -4,073.14 | 0.46 | 0.38 |
