# Supplementary material for: GeoSES: A socioeconomic index for health and social research in Brazil
Source: PLoS One. 2020 Apr 29;15(4):e0232074. doi: 10.1371/journal.pone.0232074 (PMC7190143; doi:10.1371/journal.pone.0232074)
Supplement: S2 Table — (DOCX) [file pone.0232074.s006.docx]

**S1 Table.** Geographically Weighted Regression results of models for relative risk of causes of deaths (from 5 to 74 years old) in Brazil due to interventions at the Brazilian Health System in Brazil (2013 to 2017).

| **Model** | **Residual**  **Squares** | **Effective**  **Number** | **Sigma** | **AICc** | **R^2^** | **R^2^**  **Adjusted** |
| --- | --- | --- | --- | --- | --- | --- |
| Model 1  GeoSES-IM | 4.08 | 58.19 | 0.13 | -357.89 | 0.73 | 0.67 |
| Model 2  GeoSES-IM/education | 4.41 | 56.32 | 0.13 | -338.72 | 0.71 | 0.65 |
| Model 3  GeoSES-IM/mobility | 5.30 | 58.91 | 0.15 | -276.01 | 0.65 | 0.57 |
| Model 4  GeoSES-IM/poverty | 4.56 | 62.99 | 0.14 | -313.28 | 0.70 | 0.63 |
| Model 5  GeoSES-IM/deprivation | 4.02 | 56.96 | 0.13 | -297.72 | 0.67 | 0.59 |
| Model 6  GeoSES-IM/wealth | 4.86 | 53.79 | 0.14 | -313.86 | 0.68 | 0.62 |
| Model 7  GeoSES-IM/income | 4.46 | 57.04 | 0.13 | -333.42 | 0.71 | 0.64 |
| Model 8  GeoSES-IM/segregation | 4.36 | 58.17 | 0.13 | -338.15 | 0.72 | 0.65 |
